# Heritable Variation in Garter Snake Color Patterns in Postglacial Populations

**DOI:** 10.1371/journal.pone.0024199

**Published:** 2011-09-14

**Authors:** Michael F. Westphal, Jodi L. Massie, Joanna M. Bronkema, Brian E. Smith, Theodore J. Morgan

**Affiliations:** 1 Division of Biology and The Ecological Genomics Institute, Kansas State University, Manhattan, Kansas, United States of America; 2 Department of Biology, Black Hills State University, Spearfish, South Dakota, United States of America; 3 Earlham College, Richmond, Indiana, United States of America; University of Western Ontario, Canada

## Abstract

Global climate change is expected to trigger northward shifts in the ranges of natural populations of plants and animals, with subsequent effects on intraspecific genetic diversity. Investigating how genetic diversity is patterned among populations that arose following the last Ice Age is a promising method for understanding the potential future effects of climate change. Theoretical and empirical work has suggested that overall genetic diversity can decrease in colonial populations following rapid expansion into postglacial landscapes, with potential negative effects on the ability of populations to adapt to new environmental regimes. The crucial measure of this genetic variation and a population's overall adaptability is the heritable variation in phenotypic traits, as it is this variation that mediates the rate and direction of a population's multigenerational response to selection. Using two large full-sib quantitative genetic studies (*N_Manitoba_* = 144; *N_South Dakota_* = 653) and a smaller phenotypic analysis from Kansas (*N_Kansas_* = 44), we compared mean levels of pigmentation, genetic variation and heritability in three pigmentation traits among populations of the common garter snake, *Thamnophis sirtalis*, along a north-south gradient, including a postglacial northern population and a putative southern refuge population. Counter to our expectations, we found that genetic variance and heritability for the three pigmentation traits were the same or higher in the postglacial population than in the southern population.

## Introduction

One of the primary challenges facing contemporary ecological and evolutionary research is predicting the potential effects of global climate change on populations across broad latitudinal ranges. Of recent concern has been the theoretical prediction that populations expanding into northern climates will experience a rapid loss of genetic diversity [1]–[6], which may in turn restrict the ability of populations to adapt to new selection regimes and novel stressors [7]–[10]. The current global warming event is comparable to past episodes of glacial retreat [11]. Specifically, the rate of air temperature increase in the early Holocene and the concomitant deglaciation are thought to be similar to current trends on the 100-year scale [12], [13]. Given these similarities, a productive natural experiment for exploring the genetic consequences of rapid colonization after a global warming event is to survey the current genetic makeup of populations that have undergone a rapid expansion following the retreat of glacial ice sheets 10,000 to 12,000 years ago [14], [15].

The primary hypothesis for how genetic diversity is patterned across the postglacial landscape states that populations founded after a postglacial expansion should have less genetic diversity as a result of population bottlenecks and/or new selection pressures, resulting in reduced effective population sizes and thus less genetic diversity relative to their historical populations outside the glacial barrier [16], [17]. Many studies have evaluated this hypothesis by comparing genetic diversity along latitudinal gradients spanning the glacial boundaries for various species using neutral genetic markers, including allozymes [18], [4], [7], [19], [20], mitochondrial markers [19], [21], [22], and microsatellite loci [10], [22], [23]. Overall, these neutral marker studies have supported the hypothesis that a loss of genetic diversity is associated with the expansion of populations in a postglacial landscape.

Although these studies provide a compelling picture of a population's genetic diversity following postglacial colonization, the true metric of a population's adaptive ability is not neutral genetic diversity per se, but rather heritable genetic variation. This is because heritable genetic variation mediates the rate and direction of population-scale responses to selection [24]–[26]. The few studies that have quantified heritable variation within and among postglacial populations have suggested that heritability of ecologically relevant traits may increase, rather than decrease, following rapid population expansion [23], [27]. Hypotheses to explain such an increase in heritability following postglacial colonization include: increased effects of dominance in small colonizing populations [27], [28]; the admixing of colonists from different refugia populations [22]; as well as the potential for directional selection to break down epistasis [20], [21], [23] and expose heritable variation.

To better understand the consequences of postglacial colonization on heritable genetic variation, we quantified levels of genetic variation in pigmentation traits [29] from two populations of common garter snake, *Thamnophis sirtalis*, in South Dakota, United States and Manitoba, Canada. The Manitoba population is located in the Interlake near the town of Vogar. The South Dakota population is located at Lake Traverse on the South Dakota-Minnesota border, approximately 600 km due south of the Manitoba site, and represents the southern extremis of the Laurentide ice sheet, which retreated approximately 12,000 years before present [30]. To assess potential clinal variation we analyzed phenotypic data alone from a smaller dataset from a population in Douglas County in eastern Kansas approximately 500 km due south of the South Dakota population. Pathways of garter snake postglacial migration in the region reconstructed from patterns of mitochondrial variation suggest that northern populations likely originated from populations directly to the south [31].

We focused on pigmentation phenotypes for our study because garter snake color traits are scored easily and are thought to be of adaptive importance in natural populations [32], [33]. Additionally, molecular phylogenic analysis of *T. sirtalis* has found a lack of concordance between neutral molecular trees and those based on color pattern, further suggesting a role for selection in pigmentation evolution [34]. Garter snake color patterns are important for predator avoidance, thermoregulation, and other important ecological functions [35], [36]. In this study, we examined two correlated pigmentation traits and a composite measure of the phenotypes. The first trait is the average area of dorsolateral blotches on individual snakes; the second trait is the pigmentation area of the dorsolateral blotch (the area covered by red pigment), while the composite trait is the ratio of pigmentation area of the blotch to the average area of the dorsolateral blotches.

We chose *Thamnophis sirtalis* because it is one of only a few reptile species to colonize extreme northern latitudes [37]; it has been the subject of extensive ecological, physiological, and genetic research [31], [38]–[42]; and because garter snakes have proven to be tractable organisms in quantitative genetic studies [32], [43]–[46]. Additionally, prior molecular studies (allozyme and mitochondrial) have documented the expected low genetic diversity in northern populations of *T. sirtalis* [31], [38], [47], [48], e.g. that all Manitoba populations have only a single mitochondrial haplotype at the cytochrome *b* locus [47].

The primary question we address within this multiple population framework is: Does heritability of adaptive pigmentation traits in the common garter snake decline at northern latitudes? The results of our study suggest that heritability of the three pigment traits is not significantly lower in the postglacial population. The data presented herein further shed light on the effect of rapid colonization on heritable variation in natural populations.

## Results

### Repeatability

Pearson product-moment correlation coefficients for between-observer scores for this sample were 0.926 (*P*<0.0001) for blotch area and 0.910 (*P*<0.0001) for pigment area, both of which are as high, or higher, than repeatability values considered acceptable in previous quantitative genetic studies of garter snakes [42].

### Trait means

Adult phenotypic data was collected from both male (n = 139) and female (n = 44) snakes at the Manitoba site. At the South Dakota site, females were far more numerous than males on the date of collection and the sample was therefore skewed towards females (51 females, 5 males). The Kansas sample had approximately equal representation of sexes (24 females, 20 males). Blotch area and pigment area were normally distributed for all populations. Distribution of the ratio values tended to be skewed to the right, but were nonetheless amenable to testing given the procedures used, which are generally robust to minor deviations from normality. Two-way ANOVAs with main effects of sex and population revealed no differences between sexes for blotch area (*F_1,559_* = 0.77, *P* = 0.38), pigment area (*F_1,561_* = 0.18, *P* = 0.68) or ratio (*F_1,560_* = 2.44, *P* = 0.12). Furthermore, no sex-by-population effect was detected for any trait (blotch area *F_1,559_* = 0.61, *P* = 0.43; pigment area *F_1,561_* = 1.79, *P* = 0.18; ratio *F_1,560_* = 1.72, *P* = 0.19). Because we saw no difference in trait means between sexes, males and females were pooled in all subsequent analyses. All phenotypic color estimates are in units of blotch-to-scale area, i.e. the proportion of integument that was white or red relative to the immediately adjacent dermal scute.

All trait means for adults were significantly higher in the South Dakota population relative to the Manitoba population (*P*<0.001, Tukey). However, the Kansas population had higher means for both blotch area and pigment area than the South Dakota sample (*P*<0.001, Tukey) but the composite ratio of the two pigmentation measures was significantly smaller in the Kansas population (*P*<0.001, Tukey) relative to the South Dakota population and not significantly different from the Manitoba population (*P* = 0.97, Tukey).

**Figure 1:**
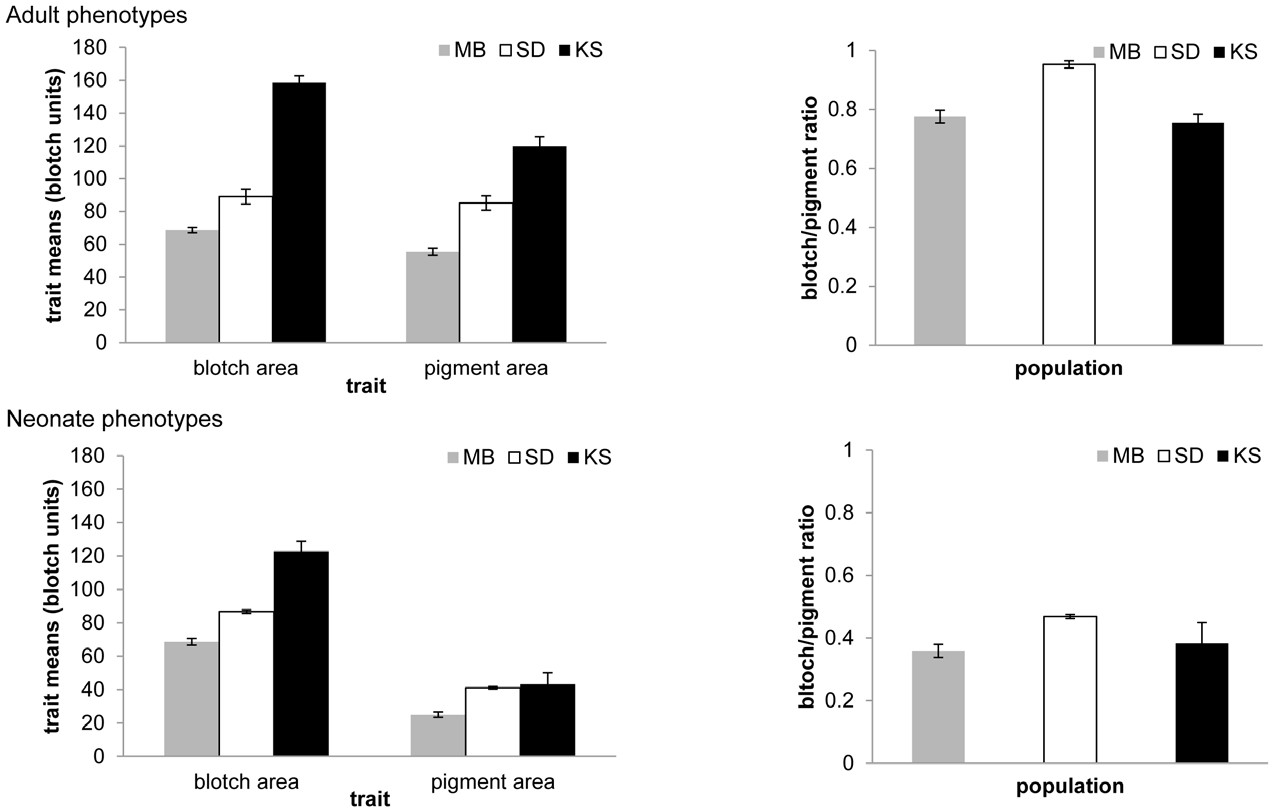
Trait means of two color traits in *Thamnophis sirtalis* from three Midwestern populations. MB = Manitoba, SD = South Dakota, KS = Kansas. Left-hand charts show means for blotch area and pigment area. Right-hand charts give means for blotch area/pigment area ratio. The upper panels are for neonates and the bottom panels are for adults. Error bars represent plus or minus one standard error of the mean. All means were significantly different from one another at or below the *P* = 0.0001 level except for the KS-MB pigment comparison for pigment (P<0.0003) and the KS-MB ratio comparison for adults (*P* = 0.0997) and neonates (*P* = 0.83) as well as two SD-KS neonate comparisons: pigment area (*P* = 0.88) and the blotch area/pigment area ratio (*P* = 0.75).

We generated a second set of phenotypic data from 144 offspring from 25 dams originating at the Manitoba site and 653 offspring from 50 dams from the South Dakota site. For the neonate snakes all pigmentation phenotypes were smaller relative to those measured on the adults (Fig. 1). Again, all means in the South Dakota population were significantly higher relative to the Manitoba population (*P*<0.001, Tukey). Kansas neonates had significantly higher blotch area (*P*<0.001, Tukey) and pigment area than the Manitoba population (*P*<0.02, Tukey) but the blotch area/pigment area ratios were not significantly different (*P* = 0.72, Tukey). Compared to the South Dakota population, Kansas neonates had significantly higher blotch area (*P*<0.001, Tukey) but pigment area was not significantly different (*P* = 0.77, Tukey) nor was the blotch area/pigment area ratio (*P* = 0.21, Tukey).

### Genetic variances and heritabilities

**Figure 2:**
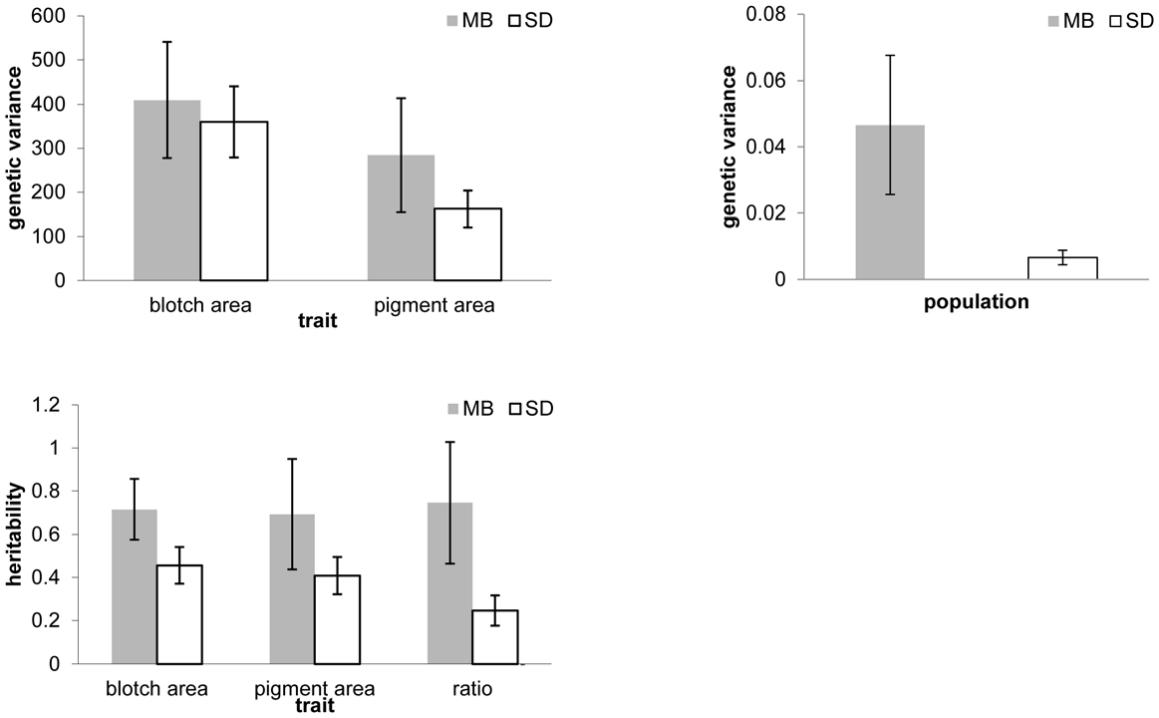
Genetic variances and heritabilities of color traits in the South Dakota and Manitoba populations of *Thamnophis sirtalis*. A. Genetic variances of blotch area and pigment area. B. Genetic variance of blotch area/pigment area ratio. C. Heritabilities of blotch area, pigment area, and blotch area/pigment area ratio. Error bars represent plus or minus one standard error of the variance as estimated by from 10,000 bootstrap replicates.

We obtained genetic variance and heritability data from two populations: 144 offspring from 25 dams originating at the Manitoba site, and 653 offspring from 50 dams from the South Dakota site. Because only small numbers of litters were available in the Kansas population, we were unable to generate usable estimates of the genetic variances and heritabilities for this population. Genetic variances and heritabilities were estimated using a full-sib ANOVA on the neonate offspring from each population. The genetic variance for blotch area was 409.62±131.64 s.e. for Manitoba and 360.07±80.96 s.e. for South Dakota; genetic variance for pigment area was 284.44±129.69 s.e. for Manitoba and 162.35±41.64 s.e. for South Dakota; genetic variance for blotch area/pigment area ratio was 0.0466±0.021 s.e. for Manitoba and 0.0067±0.002 s.e. for South Dakota (Fig. 2). Heritabilities for blotch area were 0.72±0.14 s.e. for the Manitoba population and 0.46±0.085 s.e. for the South Dakota population, while heritabilities for pigment area were 0.69±0.26 s.e. for the Manitoba population and 0.41±0.087 s.e. for the South Dakota population (Fig. 2). Heritabilities for the blotch area/pigment area ratio were 0.75±0.28 s.e. for the Manitoba population and 0.25±0.07 s.e. for the South Dakota population.

A common issue in the comparison of genetic variance estimates among populations using a quantitative genetic design is achieving statistical precision that allows the genetic variance and heritability to be bounded away from zero, and also allows those parameters to be bounded away from the population to which it is being compared [49]. Our design is robust in that we have multiple litters with hundreds of individuals from each population, thus for most of the within-population genetic estimates the 95% confidence intervals are easily bounded away from zero. However, we are unable to bound the estimates from each population away from one another based on their 95% confidence intervals (Table 1). Although within-population significance testing of quantitative genetic parameters is feasible in wild populations, comparing the same parameters among populations is difficult given the extremely large numbers of litters required [50], [51]. The fact that our estimates overlap could therefore be a consequence of a lack of statistical power, or could reflect areal-world equivalence between the populations. That said, given the robustness of the within-population estimates, our data still strongly contradicts a reduction in genetic variance in the recently colonized Manitoba population relative to the South Dakota population (Fig. 2 and Table 1).

**Table 1:** Genetic variances and heritabilities for garter snake color pattern traits.

Parameter	Location	Blotch Area (C.I.)	Pigment Area (C.I.)	Ratio (C.I.)
Genetic Variance	South Dakota	**360.07** (201.39–518.75)	**162.35** (80.72–243.98)	**0.0067** (0.003–0.011)
	Manitoba	**409.62** (151.61–667.63)	**284.44** (30.25–538.63)	**0.0466** (0.005–0.087)
Heritability	South Dakota	**0.46** (0.293–0.627)	**0.41** (0.24–0.580)	**0.25** (0.236–0.264)
	Manitoba	**0.72** (0.446–0.994)	**0.69** (0.180–1.00)	**0.75** (0.201–1.00)

Point estimates and 95% confidence intervals of genetic variances and heritability estimates for blotch area, pigmentation area, and the ratio of pigmentation area to blotch area for the Manitoba and South Dakota populations.

## Discussion

In the present study we evaluate levels of heritable variation in pigmentation between two geographically distinct populations of *T. sirtalis* on either side of the boundary of the Laurentide ice sheet, which retreated approximately 12,000 years ago [30]. We also examine a third population in less depth to gain insight into potential latitudinal trends in trait divergence. We explore the hypothesis that postglacial populations should exhibit reduced levels of genetic variation as a result of population bottlenecks and/or novel selection pressures on these populations relative to the historical populations outside the glacial boundary [16], [17]. We found two interesting patterns from this among-population study. First, there is no evidence of a decline in heritable variation for the pigmentation traits in question in the northern population. Rather, we found heritabilities to be equal in the northern population relative to the southern population (Fig. 2). Secondly, we detect a significant geographic pattern in size and coloration of the dorsolateral blotches of both neonate and adult snakes moving from southern to northern populations (Fig. 1).

Phenotypes that are adaptive and contribute to responses to environmental stresses that vary with latitude often display clinal patterns in nature [52]. We found that dorsolateral pigmentation traits (i.e., blotch area and pigment area) in *T. sirtalis* also display patterns of among-population variation consistent with a phenotypic cline (Fig. 1). That is, southern populations express significantly larger mean dorsolateral blotch area compared to populations to the north (i.e., Kansas blotch area>South Dakota blotch area>Manitoba blotch area; Fig. 1). Furthermore, this pattern is robust regardless of age (i.e., neonate or adult), which is consistent with the single population ontogenetic effects demonstrated by [46]. Although our data do not allow us to directly identify the mechanism driving this latitudinal pattern, a likely candidate variable is the difference in the thermal regimes experienced by southern to northern populations. Dorsolateral blotches are thought to function in both thermoregulation and predator startle response behavior [29], [36]. Reduction in blotch area results in a concomitant increase in the area of the integument covered by melanophores, and would be expected to increase thermoregulatory efficiency [36], an important adaptive trait in an ectotherm living in the extreme north. Because reduction in blotch area might be maladaptive with regards to predation, and because high expression of red pigment might be maladaptive with respect to thermoregulation, an evolutionary tradeoff may be playing a role in the maintenance of standing variation in pigmentation. The extent to which dorsolateral blotching mediates an evolutionary tradeoff between predator avoidance and thermoregulation deserves further study.

In addition to the pattern observed in the mean phenotypes among populations we also evaluated how heritable genetic variation is patterned amongst the South Dakota population on the southern extremis of the Laurentide ice sheet, and the Manitoba population. We found heritability to be equal in the northern population relative to the southern population. There are multiple hypotheses to explain this pattern of increased variation in postglacial populations, including population admixture or a modification of the genetic architectures underlying the traits after colonization.

In the population admixture hypothesis, genetic variation is increased as a result of genetic variation being introduced from colonists from multiple separate glacial refugia [22]. The populations in question are located in a broad zone of contact between wholly red-pigmented subspecies in the west and wholly white-pigmented in the east. In Manitoba, the zone of contact roughly coincides with the boundaries of glacial Lake Agassiz, which blanketed the northern Midwest during the glacial retreat from 12,000 to 8,000 years before present [53]. Even as the Laurentide ice sheet melted away, Lake Agassiz would have nonetheless precluded colonization of terrestrial sites. It is possible that garter snakes colonized ever northward along the east and west shores of the lake from their respective sides of the contact zone as the ice sheet retreated. Lake Agassiz drained over a very short time period about 8,000 years before present, at which time snakes might have rapidly colonized the new landscape from both the east and west, resulting in a patchwork of new populations representing different frequencies of colonists.

We know of two sources of evidence that argue against the population admixture hypothesis. Rye explored the hypothesis that Manitoba populations were hybrid products of eastern and western clades [47]. Her mitochondrial data suggest that Manitoba snakes are allied with western groups and show no affinity with eastern clades. Moreover, she delineated a mitochondrial contact zone 800 kilometers to the east of the Vogar den, suggesting that admixture with eastern populations was unlikely. Additionally, Westphal and Morgan found a developmental basis for the white/red color variation found at the Manitoba site [46]. Manitoba *T. sirtalis* were found to express significantly more white blotch area (relative to pigmented blotch area) at birth than adults from the same population, and furthermore were found to increase in red pigmentation during their early development. The South Dakota and Kansas neonates were also found to have reduced pigmentation relative to the adult samples from their respective populations. Although suggestive, neither the Rye [47] or Westphal and Morgan [46] results absolutely refute admixture as a root cause of the increase in heritability. Until further molecular work clarifies the historical dynamics of the contact zone between western and eastern clades of *T. sirtalis*, we cannot completely rule out the admixture hypothesis.

An alternative explanation for increased genetic variation in postglacial populations is the rearrangement of genetic architectures in postglacial populations [27]. Under this scenario, populations that show low heritabilities as a result of stabilizing selection in the source population become subject to drift and founder effects in the colonial population, which in turn reduces epistasis, increases dominance effects, and ultimately exposes variability that was masked in the source population. The founder effects combined with novel selection regimes can also increase genetic variability in the short term by favoring new allelic combinations [10], [20] and thus inflating heritable variation.

Where the strength of selection itself is reduced, heritabilities can be higher than under more stringent selection regimes. For example, documented correlations between quality of environment and heritability suggest that heritabilities are lower in poor environments, e.g., where selection is stronger [9]. We have therefore at least two competing selection-based hypotheses for the increased heritability of the traits in question. 1. Reduced selection. Given the paucity of competing reptile species, it may be that *T. sirtalis* in Manitoba has experienced a more favorable environment since colonization, with a concomitant decrease in natural selection and increase in heritability. 2. New selection pressures. New selection regimes in a novel environment have exposed hidden variation or favored new allelic combinations.

Although genetic diversity is expected to decrease with distance from the founding population during a stepping-stone colonization event, other factors can rescue genetic diversity. For example, high gene flow between neighboring populations can maintain allelic diversity [54]. In addition, high environmental heterogeneity can reduce genetic diversity in a colonial deme [54]. Because the Manitoba landscape is relatively non-heterogeneous (i.e. has virtually no elevational variation and essentially uniform habitat of aspen parkland), the lack of environmental heterogeneity may have contributed to the high heritability we detected. Finally, new mutations or rare alleles can go to high frequency through a founder event called “surfing,” which occurs at the leading edge of a wave of migration [54]. Due to the rapidity with which the postglacial expansion likely occurred, surfing is a valid hypothesis for the higher heritability of the Manitoba population. Regardless of the specific mechanism, a complex model of colonization with or without selection is likely necessary to explain the results of the present study.

Although at present we are unable to convincingly support or refute any explanation for an increase in genetic variance of three color traits in the Manitoba population, our results suggest that, although genetic diversity at some loci may be reduced in postglacial populations of *Thamnophis sirtalis* [38], heritable variation underlying ecologically relevant phenotypic traits may actually be higher than in the founding populations. Our results are consistent with the few previous studies that have examined heritable variation in postglacial populations [23], [27]. An important focus of future research will be to obtain robust genetic data from both populations to better assess neutral genetic variation in both regions. Analysis of microsatellite loci for *T. sirtalis* has been performed in other regions [55] and is a practical next step. Studies of Fst/Qst can address the role of selection over the postglacial landscape and will be forthcoming from the present authors. Finally, forthcoming fine-scale phylogenetic studies are expected to better resolve the issue of historical admixture in the Manitoba population. Because heritable variation can buffer populations against extirpation by allowing them increased capability to respond to selection events [7], [56], it is important to understand long-term effects on heritable variation stemming from rapid range expansion if we are to make informed decisions on preserving biodiversity in the face of global climate change.

## Materials and Methods

### Ethics statement

All work was conducted in strict accordance with the US Public Health Service (PHS) *Policy on the Humane Care and Use of Laboratory Animals*, the US Department of Agriculture's (USDA) Animal Welfare Act & Regulations (9CFR Chapter 1, 2.31), and the United States Government Principles for the Utilization and Care of Vertebrate Animals Used in Research, Teaching and Testing. The work was approved by the respective Institutional Animal Care and Use Committees (IACUCs) at Oregon State University (approval # 3175); Kansas State University (approval #2676); and Black Hills State University (approval #A-008-004).

### Field collection

**Figure 3:**
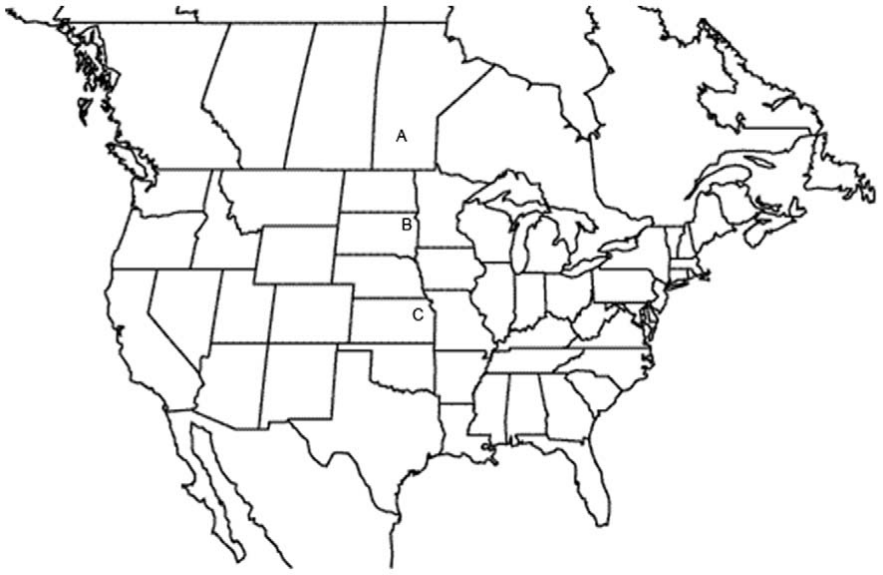
Map of collecting localities. A = Manitoba (MB), B = South Dakota (SD), C = Kansas (KS).

25 gravid female snakes were collected from a single hibernaculum near Vogar, Manitoba (Latitude: N 50° 56.934, Longitude: W 98° 34.572). A sample of 173 adult snakes (139 male, 44 female) was also collected at the Manitoba site for phenotypic scoring. 50 gravid females were collected from a single hibernaculum on the shore of Lake Traverse near Sisseton, South Dakota (Latitude: N 45° 40.528, Longitude: W 96° 43.747). Two additional gravid females were collected from a single location in Douglas County, Kansas (Latitude: N 38° 56.667, Longitude: W 95° 4.829) (see map, Fig. 3), as well as a sample of 44 adult snakes (20 males, 24 females) for phenotypic scoring. Gravid snakes were placed in breathable cloth sacks and transported to husbandry facilities. The Vogar, Manitoba sample was collected in May 2003; the Sisseton, South Dakota and Douglas, Kansas samples were collected in June 2008. We acknowledge that the temporal gap in the sampling of each population is not ideal. However, multiple years of empirical observations by MFW suggest temporal stability of the phenotypic means and variances at the Vogar, Manitoba site. Collection times at all three sites coincided with the advent of breeding season prior to dispersal to foraging habitat. Moreover, seasonal effects on these traits are likely limited, because unlike other squamates, snakes do not exhibit seasonal changes in coloration [57]. Due to the large numbers of snakes available at the Manitoba sites, specimens there were collected by grab sampling of large groups of snakes that were either basking or in mating aggregations. The South Dakota snakes were collected as they emerged from the den. The Kansas snakes were collected under cover boards within 100 m of each other during a one-day visit.

### Rearing

Female snakes were housed in dedicated facilities at Oregon State University (Manitoba sample) and Black Hills State University (South Dakota sample) and were reared under identical environmental and feeding regimes [46]. The two Kansas litters were obtained from wild-caught dams, which were reared to parturition by a private breeder under the close supervision of MFW. Prior research on the color traits analyzed below demonstrated that individuals born and reared in a common garden laboratory environment express coloration values consistent with samples from the populations of origin [46]. This concordance strongly indicates that the traits in question are stable with regard to the modest environmental variation that might be present among rearing facilities [46]. Therefore, given the similar environmental and feeding regimes at the three facilities, confounding environmental effects were not expected to arise from minor variations in rearing conditions. Nonetheless, it is widely acknowledged that heritability and genetic variance are environmentally dependent [25]. Thus we cannot definitively rule out environmental effects of breeding facility on our population comparisons, but the magnitude of such effects are likely small enough to permit such comparisons. Gravid females were retained until their litters were born. Within three days of parturition, neonates were weighed, measured snout-to-vent, and scored for color traits associated with the distinctive white and red blotches expressed in the dorsolateral region as in [46].

### Trait scoring

We used a standardized scoring system, described in [46]. The system quantifies both the average size of dorsolateral blotches and the extent to which red pigment is present in the otherwise white blotch. The final dataset is composed of two variables; blotch area, which measures the total area of blotch (irrespective of pigment type) while the pigment area measures the total area of blotch that was pigmented red. All phenotypic color estimates are in units of blotch-to-scale area, i.e. the proportion of integument that was white or red relative to the immediately adjacent dermal scute (Figure S1). Following [46] we calculated a composite trait, which was the ratio of pigmentation area to blotch area. We included this trait in the analysis because the two component traits are genetically correlated and the composite trait captures the phenotypic reality of the relative extent to which red pigmentation is expressed across the dorsolateral blotches. That is, for this composite trait individuals with a score of 1 have blotches that are completely red, while individuals with a score of 0 have blotches that are completely white. Color measurements, as well as snout-vent length, mass, and sex, were taken on individual neonates from each litter from all populations as well as representative wild caught adult snakes from each population. The timing of scoring in the present study corresponds to the first scoring period in [46]. Therefore the genetic variance of the traits was not expected to change appreciably within or among population samples across the 3-day scoring window.

### Repeatability

Two personnel were involved in scoring the pigmentation traits; therefore we conducted between-observer repeatability tests using 50 adult snakes from the South Dakota population. 50 adult snakes from the South Dakota population were scored independently by MFW and JM. Scorings were separated by a period of several months, but both measurements occurred during the stable portion of the pigmentation ontogeny that occurs when snakes are adults. Pearson correlation coefficients were calculated in SAS v 9.2 using the PROC CORR procedure to assess the repeatability of scoring between observers.

### Analysis

Trait means and standard errors for each population were obtained using PROC MEANS in SAS v 9.2. Trait means among populations were compared by a one-way ANOVA using PROC GLM in SAS v 9.2, followed by multiple comparisons. For the quantitative genetic analysis, we analyzed litter data from only the populations with a large number of litters, that is the Manitoba (25 litters and 144 offspring) and South Dakota populations (50 litters and 653 offspring). Population-specific genetic estimates (i.e. genetic variance and heritabilities) and their associated standard errors were calculated using h2boot [58]. Because neonates express pigment differently than adults [46] we used an age-specific full-sib ANOVA to estimate the genetic parameters, rather than parent-offspring regression. Estimates of genetic variance and heritability were therefore estimated for only neonates and not adults. We used 10,000 bootstrap replicates to estimate standard errors of the genetic parameters [58]. The full-sib model as implemented in h2boot can be found in [58]. Full-sib analysis is subject to overestimations of heritability due to the potential effects of common environment [25]. As discussed above, a previous common garden study of the same traits from radically disjunct populations (Manitoba, CA and California, USA) found them to exhibit strong environmental stability, suggesting maternal and other environmental effects were relatively minor [46]. Nonetheless we again cannot rule out the possibility that environmental factors contributed to our genetic estimates, but consider such effects unlikely to be so profound as to negate our results.

## Supporting Information

Figure S1 **System for scoring size and pigment saturation of dorsolateral blotches in** ***T. sirtalis*** **.** Images on left show among-individual variation in both the size of blotches and the extent to which red pigment saturates the blotches. Images on right focus on a portion of a blotch at one scale row. Top image receives a pigment area score of “0,” middle image receives a score of “0.5,” and bottom image receives a score of “1.” Schematic drawing at bottom depicts how blotch length is measured. Adjacent scale is assigned a length of 10 units, and the blotch is assigned a length relative to the adjacent scale in intervals of 1 unit. In the depicted example, the blotch at that scale row would receive a score of 7. Blotch widths at all scale rows are summed over three adjacent blotches to give individual blotch area score. Pigment area at each scale row is obtained by multiplying the pigment score by the blotch length. Pigment scores at each scale row are then summed over the same three adjacent blotches to give the individual pigment area score.
(DOC)
