# Supplementary figures and images for: Heritable Variation in Garter Snake Color Patterns in Postglacial Populations
Source: PLoS One. 2011 Sep 14;6(9):e24199. doi: 10.1371/journal.pone.0024199 (PMC3173445; doi:10.1371/journal.pone.0024199)

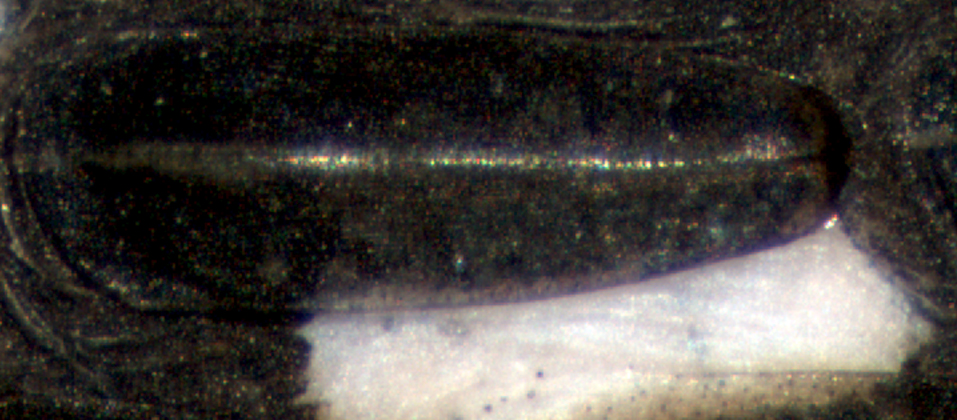

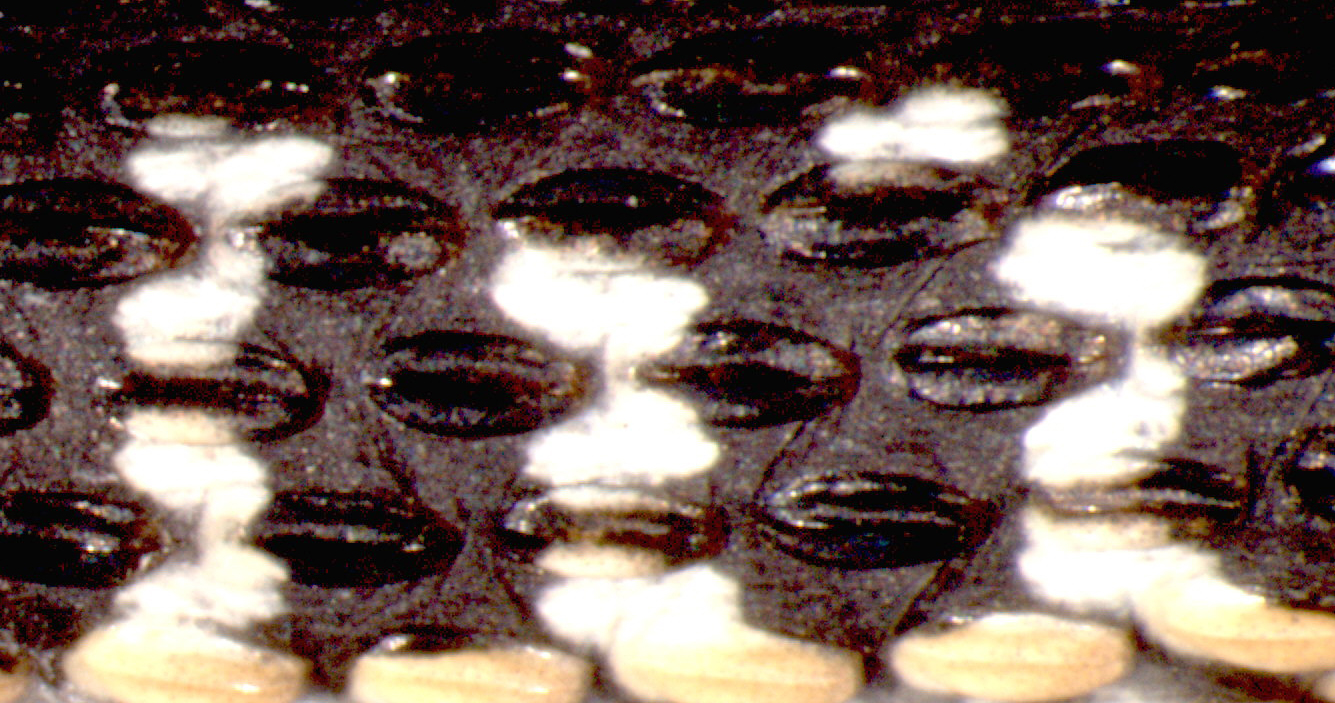


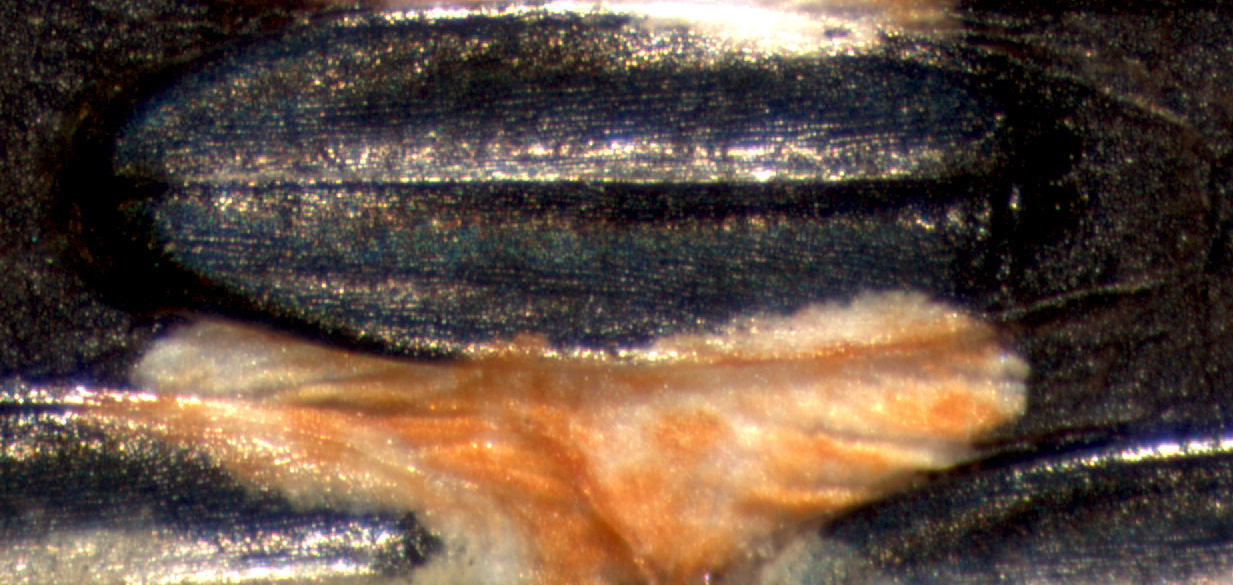

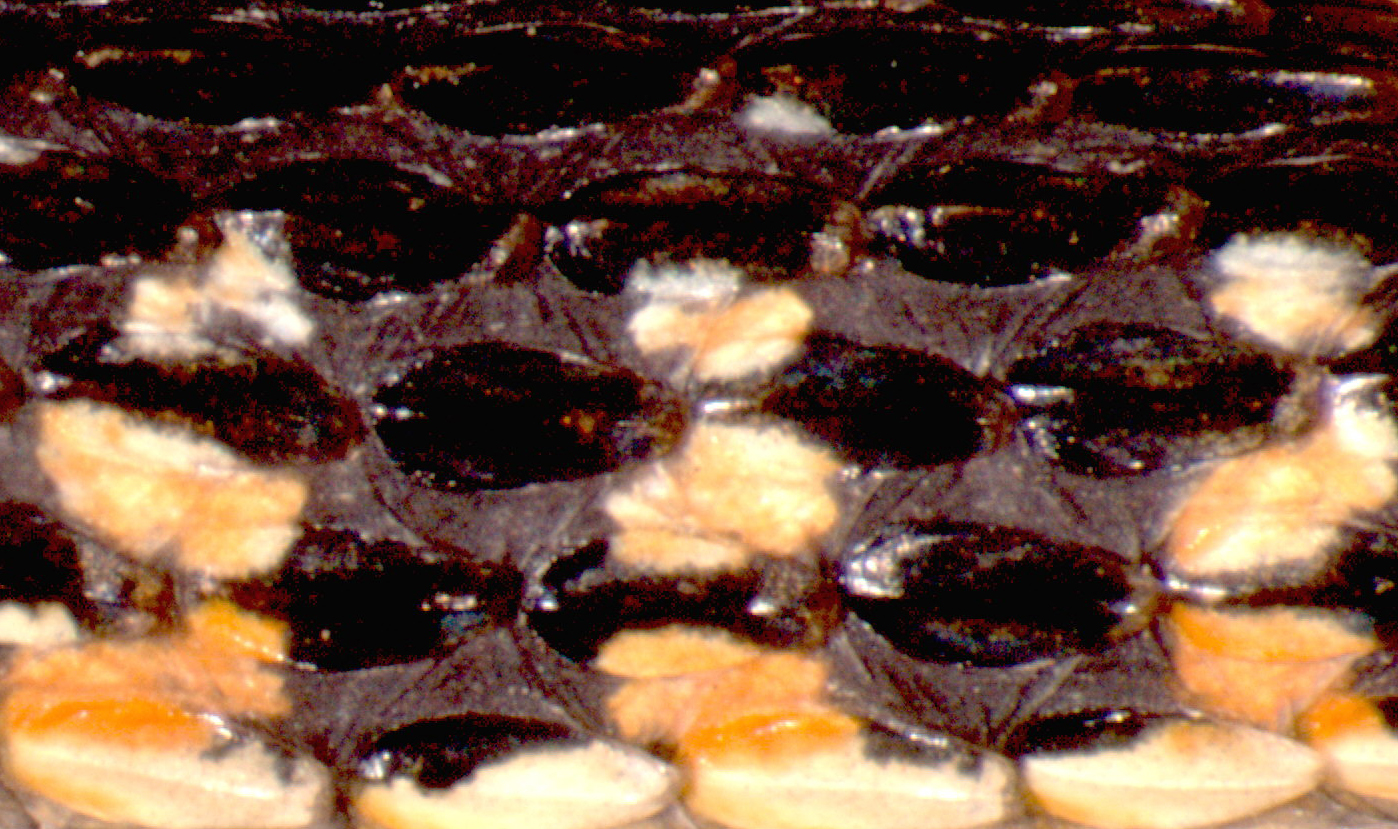


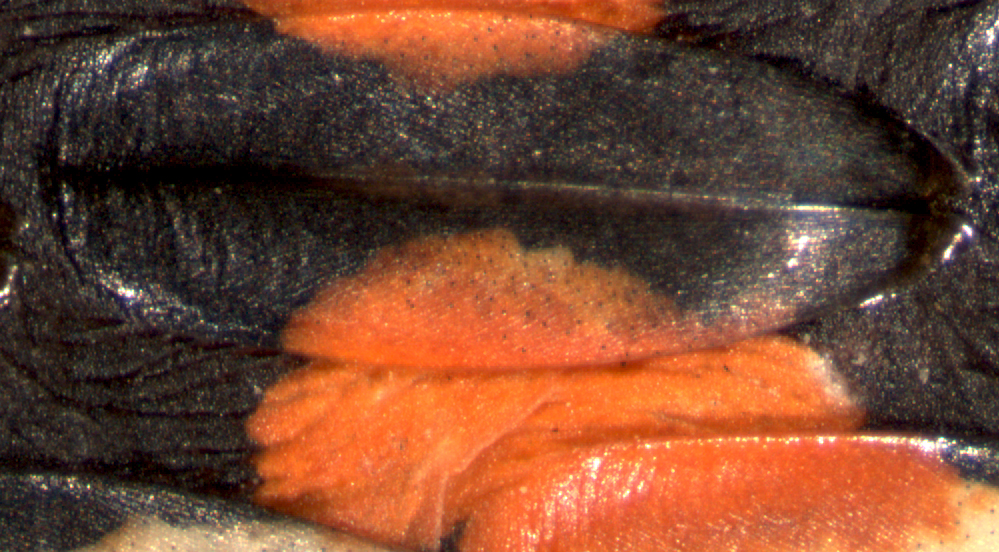

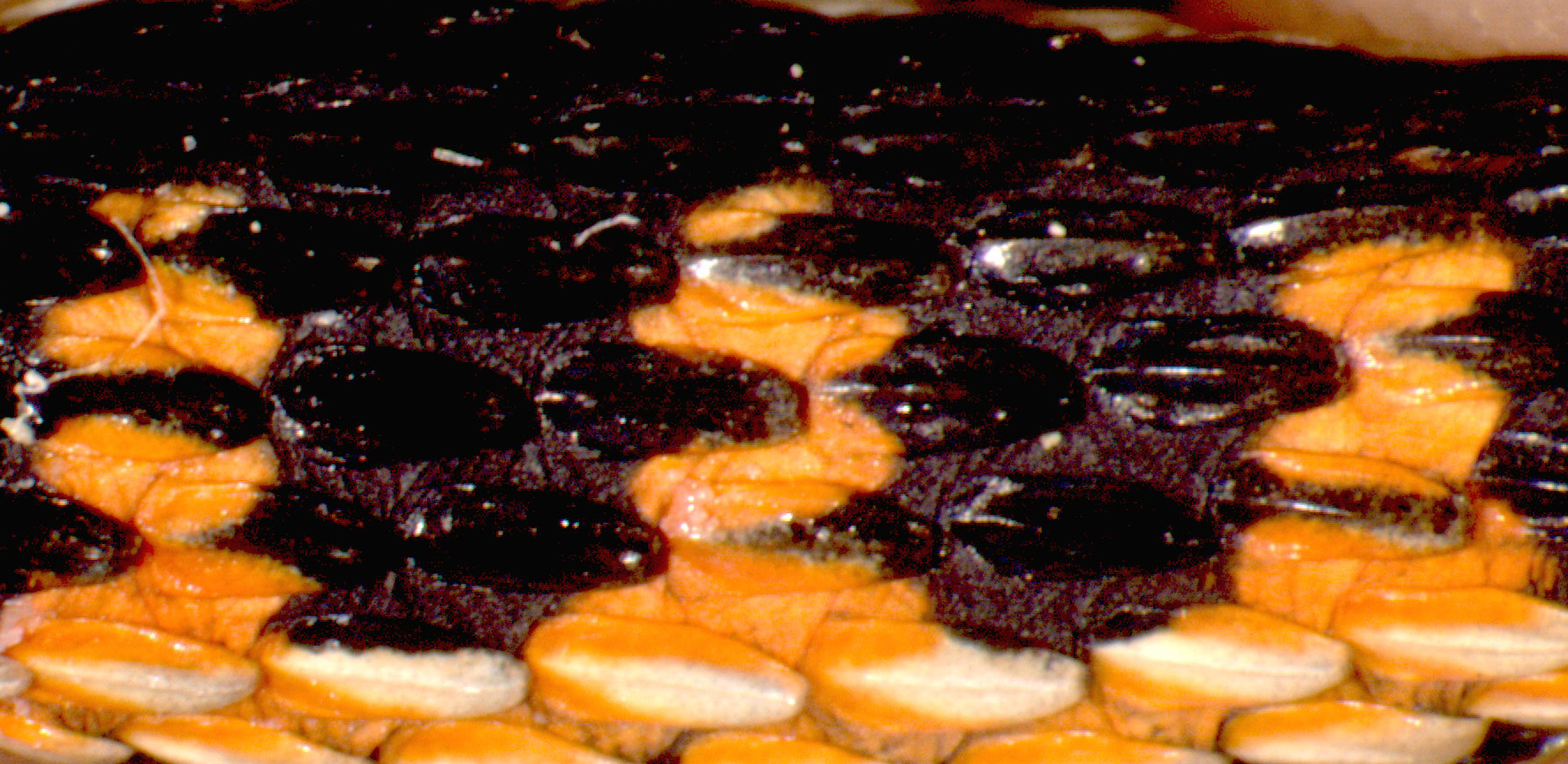


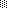


**0**

**10**

**0**

**7**

Supplement: Figure S1 — System for scoring size and pigment saturation of dorsolateral blotches in T. sirtalis . Images on left show among-individual variation in both the size of blotches and the extent to which red pigment saturates the blotches. Images on right focus on a portion of a blotch at one scale row. Top image receives a pigment area score of “0,” middle image receives a score of “0.5,” and bottom image receives a score of “1.” Schematic drawing at bottom depicts how blotch length is measured. Adjacent scale is assigned a length of 10 units, and the blotch is assigned a length relative to the adjacent scale in intervals of 1 unit. In the depicted example, the blotch at that scale row would receive a score of 7. Blotch widths at all scale rows are summed over three adjacent blotches to give individual blotch area score. Pigment area at each scale row is obtained by multiplying the pigment score by the blotch length. Pigment scores at each scale row are then summed over the same three adjacent blotches to give the individual pigment area score. (DOC) [file pone.0024199.s001.doc]
